# Primary hip arthroplasty for the treatment of alkaptonuric hip arthritis: 3- to 24-year follow-ups

**DOI:** 10.1186/s42836-019-0010-8

**Published:** 2019-10-03

**Authors:** Javahir A. Pachore, Vikram Indrajit Shah, Sachin Upadhyay, Kalpesh Shah, Ashish Sheth, Amish Kshatriya

**Affiliations:** 1 Department of Hip Arthroplasty, Shalby Hospitals, Ahmedabad, Gujarat India; 2 Department of Knee, Shalby Hospitals, Ahmedabad, India; 3 Department of Orthopaedics, NSCB Medical College, Jabalpur, MP India; 4 Department of Trauma, Joint Replacement and Minimal Invasive Surgery, Shalby Hospitals Jabalpur, Jabalpur, Madhya Pradesh India; 5 Department of Knee and Hip Arthroplasty, Shalby Hospitals, Ahmedabad, Gujarat India

**Keywords:** Total hip replacement, Alkaptonuric arthritis, Ochronosis, Harris hip score

## Abstract

**Background:**

The objective of this study is to share our experience in total hip replacement for the treatment of ochronotic hip arthritis, in particular to report how to establish the diagnosis and some tips to limit complications.

**Method:**

A cohort comprised of 10 patients (12 hips) with alkaptonuric hip arthritis. There were six men and four women with the mean age of 62.80 ± 7.57 years. All patients had a stiff spine, grossly restricted movements of hip joints, and severely limited daily routine activities. Total hip replacement was performed in all patients. The patients were evaluated at 6, 12, and 24 months after surgery, as well as every 4 years thereafter. Harris hip score was used to assess the functional outcome. The level of significance was set at *p* < 0.05.

**Results:**

The mean follow-up lasted 16.70 ± 6.82 years (3 to 24 years). At the final available follow-up, nine patients returned to work, ambulate without an orthosis, and achieve complete pain relief. Harris hip score was improved from poor to excellent. One patient died 16 years after surgery due to breast cancer. No complication relating to prosthetic failures was detected.

**Conclusion:**

Total hip replacement gives long-term satisfactory results in patients with alkaptonuric hip arthritis, resulting in comparable function of the hips in patients who undergo primary osteoarthrosis.

## Background

First described by Virchow in 1866, ochronosis is the connective tissue manifestation of alkaptonuria, an autosomal recessive mutation of the HGO gene on chromosome 3q, caused by deficiency of homogentisate 1, 2 dioxygenase activity [1]. Homogentisic acid oxidase is responsible for turnover of homogentisic acid (HGA) during the course of phenylalanine and tyrosine catabolism [2]. HGA accumulates and is polymerized into a blue-black pigment that is ultimately deposited in skin, bones, tendons, articular cartilages, synovial membranes, lungs, valves, and kidneys [3]. The accumulation eventually causes severe degeneration of the spine and peripheral joints, which may clinically lead to common arthritic disorders [4]. Alkaptonuria is a rare metabolic disorder characterized by a triad of degenerative arthritis, ochronotic pigmentation, and homogentisic aciduria, affecting one in 250,000 to 1 million people [5, 6]. Chromatographic, enzymatic or spectrophotometric determinations of HGA are confirmatory tests. Currently, there is no definitive cure for alkaptonuric ochronosis. Symptomatic treatment of the complications of alkaptonuria is the only option, including pain management, physiotherapy, chiropractic care, and instruction regarding a home exercise program. A successful treatment for tendon ruptures caused by ochronosis is primary repair [6]. High dose of vitamin C decreases urinary benzoquinone acetic acid, but has no effect on HGA excretion and, moreover, no credible studies have shown that treatment with vitamin C is clinically effective. Nitisinone, a potent inhibitor of 4-hydroxyphenylpyruvate dioxygenase, dramatically reduces production and urinary excretion of homogentisic acid [7], however, the effectiveness of Nitisinone in treating ochronosis remains unknown. Patients with alkaptonuria are usually asymptomatic, and the ochronotic arthropathy appears after the fourth decade [8]. Total replacement of hip, knee, elbow, and shoulder can alleviate pain and increase patient’s daily activities [2]. There is a paucity of studies concerning total hip replacement in the present patient population with adequate follow-up statistics.

The primary objective of the present study is to share our experience in total hip replacement (THR) for ochronotic hip arthritis, in particular to report on establishment of diagnosis and tips to decrease the complications.

## Patients and methods

The study was approved by the Institutional Ethics Committee of the hospital. Informed consent was obtained from each patient.

**Fig. 1:**
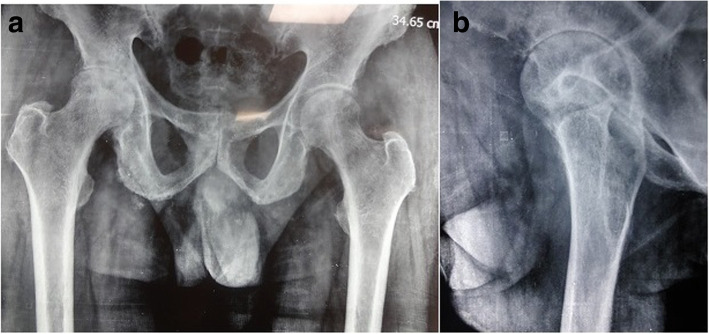
**a** & **b** Anteroposterior and lateral X-ray films of the pelvis, with both hip joints showing reduced join space with degenerative changes, irregularity, and flattening of femoral head

**Fig. 2:**
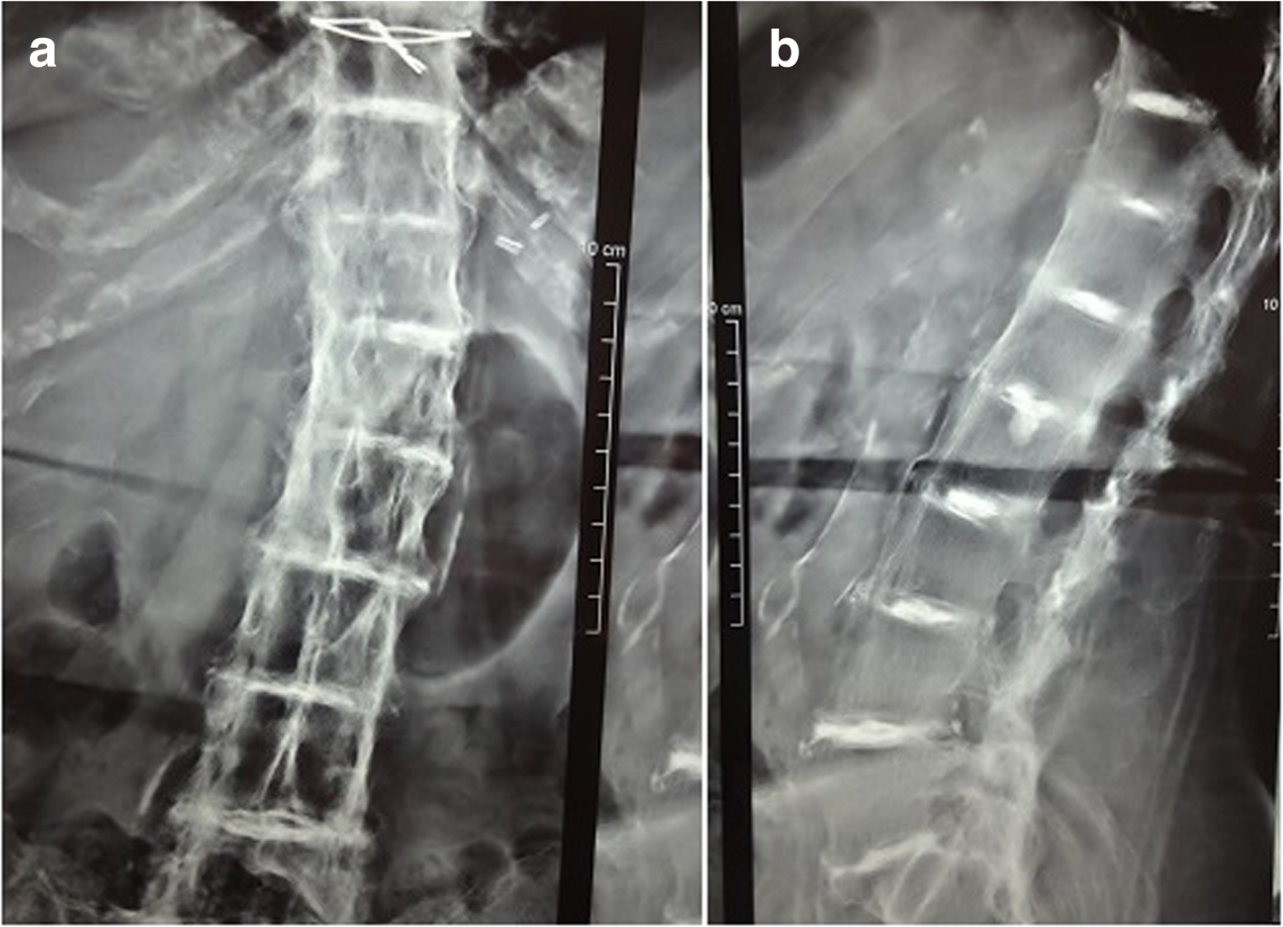
**a** & **b** Anteroposterior and lateral spine X-ray films revealed osteoporosis, flattened, and intra-discal calcification (doubling signs), and variable degrees of fusion of the vertebral bodies

A case series of 10 patients (12 hips) with alkaptonuric hip arthritis who presented to our institute were reported. There were six men and four women with the mean age of 62.80 ± 7.57 years (range, 53–80 years). Demographic variables, presentation, comorbidities, and preoperative diagnoses were recorded and analyzed (Table 1). All patients had pain in groin region for the past 4 months to several years. None of the patients was under specific treatments. Clinical, imaging, and laboratory assessments were performed in all patients. Of the 10 patients, seven patients had a family history of alkaptonuria, and eight patients had cutaneous signs of ochronosis. Nine patients had a history of blackish discoloration of urine on exposure to air. Spine examination revealed stiff spine with limited movements in all patients. On local examination, hip movements were painful and severely restricted. All patients reported gross limitation of daily routine activities and needed assisted ambulation. A dried urine spot (DUS) [9] detects HGA in all patients. Other laboratory parameters were within the normal ranges. The standard radiographs of hip showed severe joint degeneration and narrowing of the joint space with irregularity and flattening of the femoral head (Fig. 1a, b). A lateral spine radiograph revealed osteoporosis, flattened and intra-discal calcification, and variable degrees of fusion of the vertebral bodies (Fig. 2a, b). Pre-operatively, the mean Harris Hip Score was 35.00 ± 14.06. All patients were diagnosed with ochronotic hip arthritis, and underwent primary hip arthroplasty. Eight patients underwent unilateral surgery and two had bilateral procedures. In bilateral cases, the priority was given to the side with more severely affected joint.

**Table 1:** Characteristics of patients

Case	Age/sex	Duration of pain	Side of hip	Cutaneous manifestations	Urine discoloration	Family history	Spine	Comorbidities	Presentation	Last follow up
1	53/M	3 years	Left	Present	Yes	Yes	Stiff with restriction of movements	Nil	Bilateral groin pain; Disturbed sleep; needed assistance in the form of elbow crutches to walk; undergone bilateral total knee replacement 2 years back Patient was advised Right side uncemented total hip replacement.	3.7 years
2	55/M	1 year	Right	Present	Yes	Yes	Stiff with restriction of movements	Nil	Right groin pain; unable to walk for more than 5mins, sleep was disturbed	16.4 years
3	80/F	4 months	Both	Absent	Yes	No	Stiff with restriction of movements	Hypertension, Diabetes, Ischemic Heart disease, Valvular calcification, Hypothyroidism	Pain in left side of groin for past 4 months and pain in both the knees; unable to walk due to pain. Right total hip replacement was done years back.	15.7 years (Right hip) 13.7 years (left)
4	65/F	10 years	Both	Present	Yes	No	Stiff with restriction of movements	Hypertension, Diabetes	Pain in both groin, along with bilateral knee pain and back pain for past 10 years; Patient was bed ridden and disabled with pain	18.5 (Left) 16.7 (right) years (bilateral hip) patient died ~ 16 years after the second surgery
5	63/F	6 months	Left	Present	Yes	No	Stiff with restriction of movements	Hypertension, Diabetes, Hiatus Hernia, Umbilical hernia, peptic ulcer	Pain in the left groin for the past 6 months. She also had pain in both the knees and shoulder with limitation of movements.	22.5 years
6	66/F	10 months	Left	Present	Yes	Yes	Stiff with restriction of movements	Hypertension, Diabetes; Obese	Pain in both the groin for past 10 months. Left hip was more painful than the right side causing difficulty in walking and need of assistance in the form of walker to walk. Low Backache	19.2 years
7	60/M	1 years	Right	Present	Yes	Yes	Stiff with restriction of movements	Hypertension, Diabetes	Spontaneous and increasing pain in both hips, more severe on the right. Low backache with difficulty in walking	20.2 years
8	64/M	1.5 years	Right	Present	Yes	Yes	Stiff with restriction of movements	Hypertension, Diabetes	Progressive pain in right hip joint for the last 1.5 years and low back ache from the last 10 years. Orthosis assisted walking	10.5 years
9	57/M	2 years	Right	Absent	No	Yes	Stiff with restriction of movements	Nil	Pain in right groin since 2 years with marked restriction of movements.	24.1 years
10	65/M	10 months	Left	Present	Yes	Yes	Stiff with restriction of movements	Hypertension	Pain was dull aching, insidious in onset, continuous in nature more on the left side than right, interfering with his day to day activities	18.9 years

### Surgical technique

**Fig. 3:**
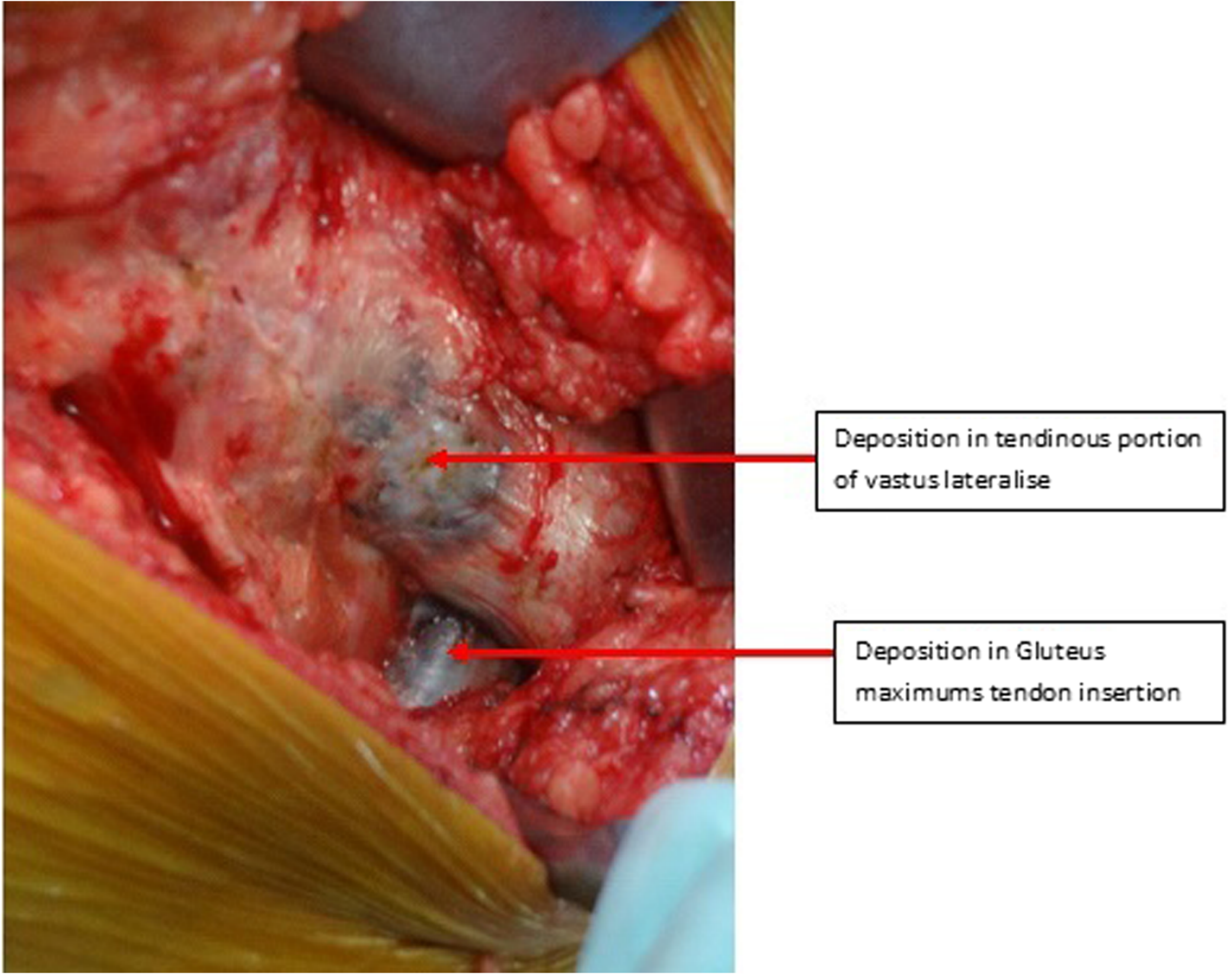
Black discoloration over the tensor fascia lata, and calcification of the hard tissues with blackish deposit over the gluteus muscles

**Fig. 4:**
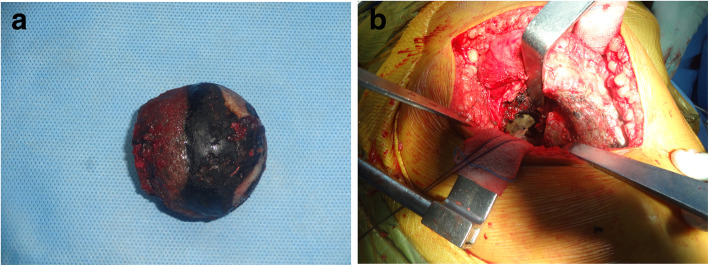
**a** A classical black painted articular cartilage of the femoral head; **b** A black painted articular cartilage of the acetabulum with sclerosis

All surgeries were performed by the same senior surgeon and the same arthroplasty team through the posterior approach with the patients in the lateral decubitus position. The incision was made over the center of the trochanteric region and then was adequately lengthened both cranially and caudally for a good exposure to avoid forceful retraction. The subcutaneous fat was incised deep to the fascia lata. We found black discoloration over the tensor fascia lata, and calcification and hard tissue at the insertion of gluteus maximus tendon with blackish deposits (Fig. 3). We found deposition of black tissues with some degrees of fragility in the gluteus medius tendon. The fascia lata was incised and the gluteus maximus tendon was bluntly splited along the direction of fibers. The trochanteric bursa overlying the external rotators was incised. The sciatic nerve was carefully protected. The short external rotators were tagged with a suture for identification and subsequent repair. These rotators were detached at their insertions onto the greater trochanter and reflected posteriorly to expose the posterior joint capsule. The capsule was found to be more fibrotic and contracted with blackish discoloration. If the capsule was hard and contracted, we usually incised the capsule from 6 o’clock to 12 o’clock to ease dislocation of the femoral head. When the hip was dislocated, we found the femoral head was covered with typical black painted articular cartilage, but the subchondral bone was not affected (Fig. 4a). The tip of the trochanter was impregnated with thick black pigments. During the preparation of the femur, great care should be taken to avoid fracturing the tip of trochanter. Preparation of the acetabular bone was difficult due to the black deposition that looked like a layer of tissue (Fig. 4b). The bone was sclerotic, hard, and difficult to ream, so as to open subchondral bone (Fig. 4b). We conducted either pre-drilling or curettage of the cup and finally reamed it to get punctate bleeding. Furthermore, owing to the sclerotic rim cup, expansion of the acetabulum was probably difficult. Therefore, additional screws were used to secure the fixation. After trailing, the final cup position was achieved within the safe zone for both anteversion and inclination. The femoral preparation was done in the routine fashion, because the femoral canal was normal. After placement of the optimal stem and femoral head, THR was completed and hip joint was reduced. Closure was done in layers under negative suction drain.

### Postoperative managements

The pigmented cartilaginous surface, bone, and soft tissues were sent for histomorphological evaluation. Our protocol was to remove the drain on the second postoperative day. The patient was discharged on the sixth postoperative day, and then given a standard rehabilitation.

### Postoperative evaluation

All patients returned for follow-ups at 6, 12, and 24 months, as well as every 4 years thereafter. Preoperatively and at the final follow-up, functional outcome evaluation was performed using HHS. Standard anteroposterior and lateral femoral X-rays were obtained to study heterotopic ossification, radiolucent lines, position of stem and subsidence, the change in the cup inclination or cup migration, and loosening of implants. All parameters were expressed as mean ± standard deviation. The differences were analyzed with student’s t test. A *p* < 0.05 was considered statistically significant.

## Results

In this series, the postoperative course was uneventful. No intraoperative complications other than increased blood loss were observed. Histopathological examination of periarticular tissues revealed that tissue pigmentation was most markedly in the deeper layers, with areas of calcification and degeneration. The synovium was thickened, inflamed, and pigmented. HGA was detected using dried urine spots [9] in all patients. In all cases, there was a macroscopic layer of black pigmented articular cartilage, which was more severe in elderly patients. Histopathological, physical, biochemical, and radiographic evaluations confirmed the diagnosis of ochronotic arthropathy. One patient died 16 years after the second hip surgery due to breast cancer. Therefore, a total of 10 hips were followed-up finally. The mean follow-up time was 16.70 ± 6.82 years (range, 3 to 24 years). At 6-month follow-up, the mean HHS improved significantly, from 35.00 ± 14.06 preoperatively to 93.20 ± 1.75 (*p* < 0.05). Radiographs showed stable prostheses in situ without any evidence of subsidence (Fig. 6a, b, c, d, e, f, g and Fig. 7a, b, c, d). The latest follow-up showed that nine patients (10 hips) went back to work and ambulated without walking aids. All patients had complete pain relief. HHS was improved from poor to excellent. Although patients had complaints pertaining to low backache, none of the patient had major complications requiring a revision surgery. We did not found complications related to prosthetic failures (Fig. 6a, b, c, d, e, f, g and Fig. 7a, b, c, d).

## Discussion

**Fig. 5:**
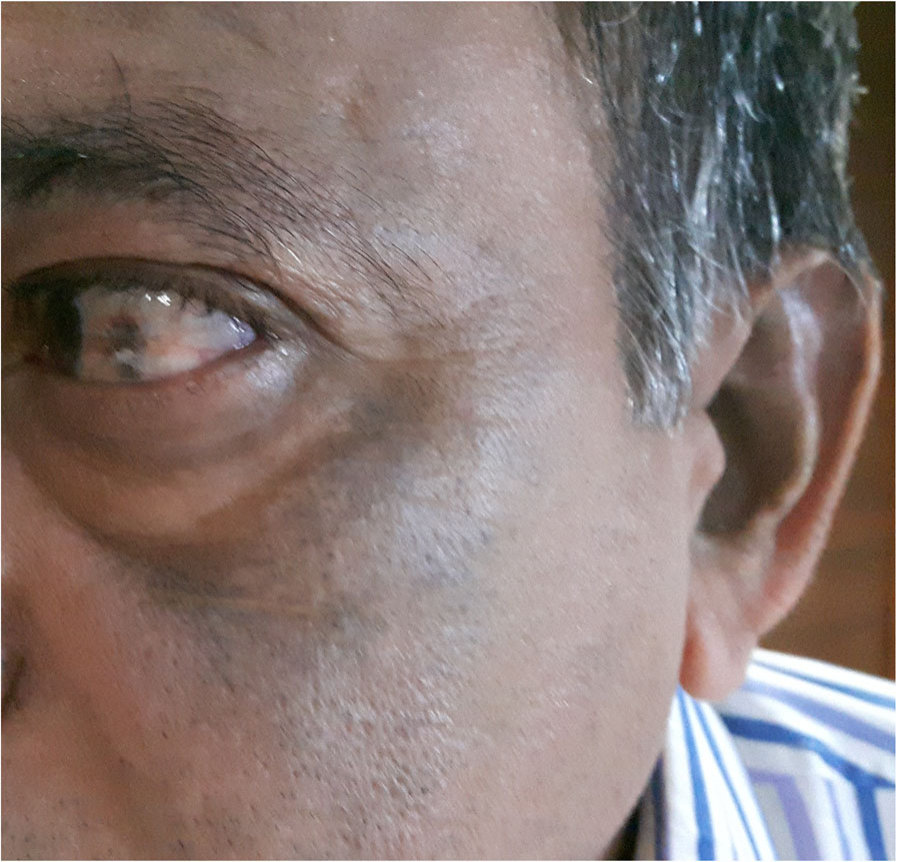
Cutaneous signs of ochronosis that include changes of the color of the sclera and ears

Alkaptonuria is a rare autosomal recessive inborn metabolic disorder of tyrosine metabolism due to deficiency of homogentisic oxidase enzyme. Alkaptonuria is characterized by excretion of homogentisic acid in urine, and deposition of oxidized homogensitate pigments in the connective tissues and articular cartilages (ochronosis). The gene for this pathological condition is present at locus 3q21–23 [10]. In ochronosis, there is deposition of pigments from homogentisic acid in all types of connective tissues, including cartilage, cardiovascular system, genitourinary system, sclera, skin, and ear cartilage [11, 12]. Urine discoloration is the first clinical manifestation of alkaptonuria, followed by color changes of the sclera and ears. Based on the detailed general examination, cutaneous signs of ochronosis include color changes of the sclera and ears which can easily be observed (Fig. 5). 80 % of cases in our series had positive association. There is a paucity of available literature concerning the occurrence of ochronosis arthropathy without ocular and cutaneous signs. Kusakabe et al. [13] showed cervical arthropathy without ocular and dermatological findings. Retrospective inquiry for the family history of alkaptonuria usually makes sense. In the present ten patients, seven had a positive family history. Patients will respond to the leading question about discoloration of urine. Ninety percent of the patients in our series had a history of blackish discoloration of urine on exposure to air.

The tendons and ligaments may also be affected because of their high collagen content. It causes inflammatory alterations resulting in rupture of the tissues [6]. Early diagnosis of ochronosis is valuable to avoid tendon ruptures. The preoperative cardiac clearance is imperative to rule out the risk of valvular calcification [12]. In our series, one patient who had a history of valvular calcification underwent aortic valve replacement and coronary artery bypass graft surgery.

Alkaptonuric arthropathy has previously been shown to be a relatively benign disease. Our evidence showed that the patients with this disease were crippled and disabled with pain. Recent overall increase in life expectancy may account for the scenario. Although alkaptonuria affects both men and women with ochronotic arthropathy, the trend is more severe and more frequent for men than for women. The result is also male preponderance (six men), which was compatible with our series [14, 15].

Patients with alkaptonuria are usually asymptomatic, and arthropathy appears after the third or fourth decade with a sudden onset of pain limiting daily routine activities [15, 16]. A late onsets is attributed to the gradual age-related decline in the renal ability to excrete homogentisic acid, resulting in a diseased association with signs and symptoms of accumulation of homogentisic acid. In about 50% of patients, alkaptonuric cases develops arthropathy [17]. Low backache precedes joint diseases. In our cases, low backache with restricted mobility was on the foreground. In most of the cases, spinal examination showed the restricted movements of the spine. Back pain and stiffness in the thoraco-lumber junction and cervical region may be the initial symptom of ochronosis. The spinal stiffness is almost like ankylosing spondylitis but the age group is different. Pain is more severe in patients with ankylosing spondylitis than in patients with ochronosis, which have an increased degeneration that may not proportionally increase severity of pain. There is a loss of cervical or lumbar lordosis. There may be kyphosis or localized scoliosis. In a few of such cases, root pain or sciatica could be the presenting or only symptom in ochronotic arthropathy. Spine radiograph shows intra-discal calcifications giving a “doubling” of the outline, which confirmed the diagnosis of ochronosis [18]. In patients with ankylosing spondylitis, there is extensive ossification of spinal ligaments with little calcification of the intervertebral discs.

Ochronotic arthropathy usually involves the large weight-bearing joints (knee and hip joints) rather than the small joints of the hand and foot [19–21]. In our series, the hip joints were severely affected. Movements of the hip joints were painful and were restricted due to the arthropathies. Similar to patients with primary osteoarthrosis, concentric reduction of the joint space is frequently seen on X-rays. However, the radiological alterations may be much less apparent than the clinical manifestations.

Intraoperatively, we noticed an increased blood loss, which may be the result of *en bloc* synovectomy of the hypertrophied synovium. The blood loss was more than what is usually observed in arthritis of other common causes. Although the hemoglobin and haematocrit levels were not decreased after surgery, we still advise to avoid total synovectomy without intraoperative bleeding control. In ochronotic cases, Cebesoy et al. [22] has recommended complete removal of the joint capsule. We speculated that complete capsular resection could increase the rate of dislocation postoperatively. Therefore, the capsule should be preserved and utilized for capsular closure. We did not find any complication with the use of the technique.

The bone quality around the hip joint affects the stability of prosthesis. During reamerization, Cebesoy et al. [22] found poor bone quality at both the acetabulum and proximal femur irrespective of the patient’s age. In our cases, we noticed the bone quality of the proximal femur was in accordance with the age of patients, but acetabulum wall was sclerotic, which was attributed to deposition of pigments in the deeper layers of articular cartilage (Fig. 4b). As a result, the cartilage loses its elasticity and become sclerotic [15]. In view of the matter, we advise to secure the cup with screws, because the sclerotic rim may impede the cup expansion.

**Fig. 6:**
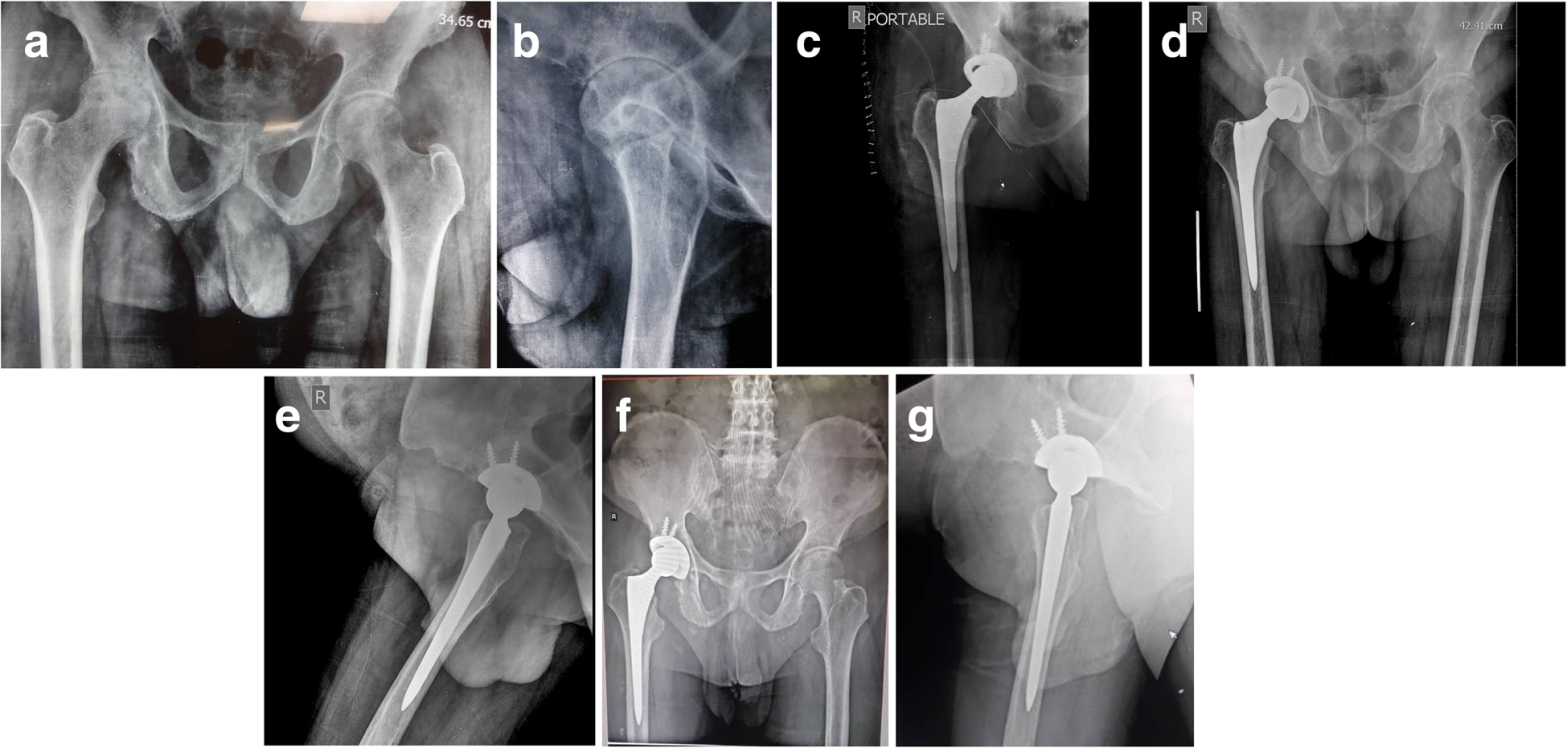
Pelvis and both hips on X-rays. **a** preoperative anteroposterior view; **b** lateral view **c** anteroposterior view immediately after surgery; **d** anteroposterior view 5 years after surgery; **e** Lateral view 5 years after surgery; **f** anteroposterior view 10.5 years after surgery; **g** Lateral view 10.5 years after surgery

**Fig. 7:**
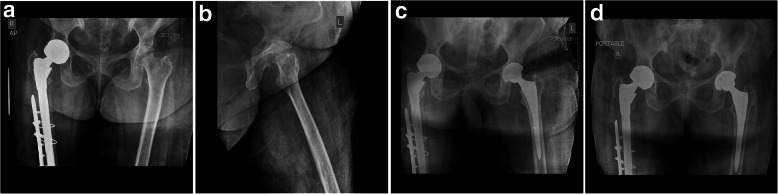
Pelvis and hips on X-rays. **a** an anteroposterior view preoperatively (left); follow-up X-rays after 2 years (uncemented ASR with S-ROM; right); **b** preoperative lateral view of left hip; **c** an anteroposterior view immediately after surgery (left) and follow-up after 2 years (right); **d** anteroposterior view at the final follow-up of 15.7 years (Uncemented ASR with S-ROM; right) and left hip (13.7 years; Pinnacle with Summit)

Before 1980, we did cemented THR because we did not had other choices, and in the rest of the hips we used cementless THR. Because the bone tissue is uncommonly involved, we were not suspecting any bone-ingrowth deficits that might affect stability of the cementless implants [15, 23]. In the present study, we didn’t observe instability, early loosening, subsidence, or protrusion problems on radiographs. Radiolucent lines, migration, or change in alignment were not observed on the acetabular socket. These factors are suggestive of stable implant with bone growth (spot welding). On femoral side, we did not find subsidence, radiolucent lines, or instability of femoral components (Fig. 6a, b, c, d, e, f, g and Fig. 7a, b, c, d). Long-term follows showed that superior pain relief and successful restoration of hip function were achieved (mean follow-up time: 16.70 ± 6.82 years; range, 3 to 24 years) in patients with ochronotic arthropathy. We therefore conclude that both the uncemented and cemented total hip had long term survivorship. Our preference is to use a cemented THR in patients with poor bone stock or osteoporotic bone. Furthermore, in view of patient’s stiff spine in present series, we strongly believe that in the early era of total hip arthroplasty in India, very little or no significance was attributed to the stiffness of spine and /or deformity of spine. We have operated on the patients in present series for a long time before the inception of concept of spinopelvic mobility and total hip arthroplasty. The concept of spinopelvic parameters/movements in relation to THR is new. So, we did not have preoperative lateral spinopelvic-hip X-rays, nor sitting or standing lateral spine X-rays. In the early years, we had intraoperatively used manual jigs and eyeballing to achieve optimal component alignment with respect to patient’s anatomy and limb length. Versions/offsets were attended independently to ensure optimal component positioning and a stable hip. In cemented hip, the principals of Charnley hip were followed to measure various angles. Recently, we used the principles of Scott Ranawat co-plannar test in uncemented surgery. Fortunately, there was no dislocation in these groups of patients, in spite of a 22-mm femoral head used.

There is no specific medical treatment for alkaptonuria, and hence all therapeutic approaches are symptomatic. In severe osteoarthrosis, total hip arthroplasty is the preferred treatment. Results from our series are consistent with the available literature [2, 22, 24–26]. The findings support reliability of THR in patients with ochronotic arthropathy. We hope the rationale behind the present series can guide the surgeons to establish the pre-operative diagnosis and to facilitate the intra-operative procedures. No complications related to implant failure were detected after total arthroplasty, and our results are compatible with patients who underwent primary osteoarthrosis.

## Conclusion

Primary hip arthroplasty is an effective and preferred treatment for alkaptonuric hip arthritis. The critical factor that directly influences the outcomes of total arthroplasty is surgeons’ acquaintance with the ochronosis. Through these cases we have attempted to provide tips pertinent to the establishment of the diagnosis of ochronosis and performance of THR for alkaptonuric hip arthritis. Those tips can help to lower surgical complications.

## Data Availability

The data that support the findings of this study are available from [Shalby Hospitals India] but restrictions apply to the availability of these data, which were used under license for the current study, and so are not publicly available. Data are however available from the authors upon reasonable request and with permission of [Shalby Hospitals India].

## Abbreviations

DUS: Dried urine spot
HGA: Homogentisic acid
HHS: Harris hip score
THR: Total hip replacement
